# Supplementary figures and images for: PPI-ID: Streamlining protein-protein interaction prediction through domain and SLiM mapping
Source: PLoS Comput Biol. 2025 Oct 16;21(10):e1013062. doi: 10.1371/journal.pcbi.1013062 (PMC12571275; doi:10.1371/journal.pcbi.1013062)

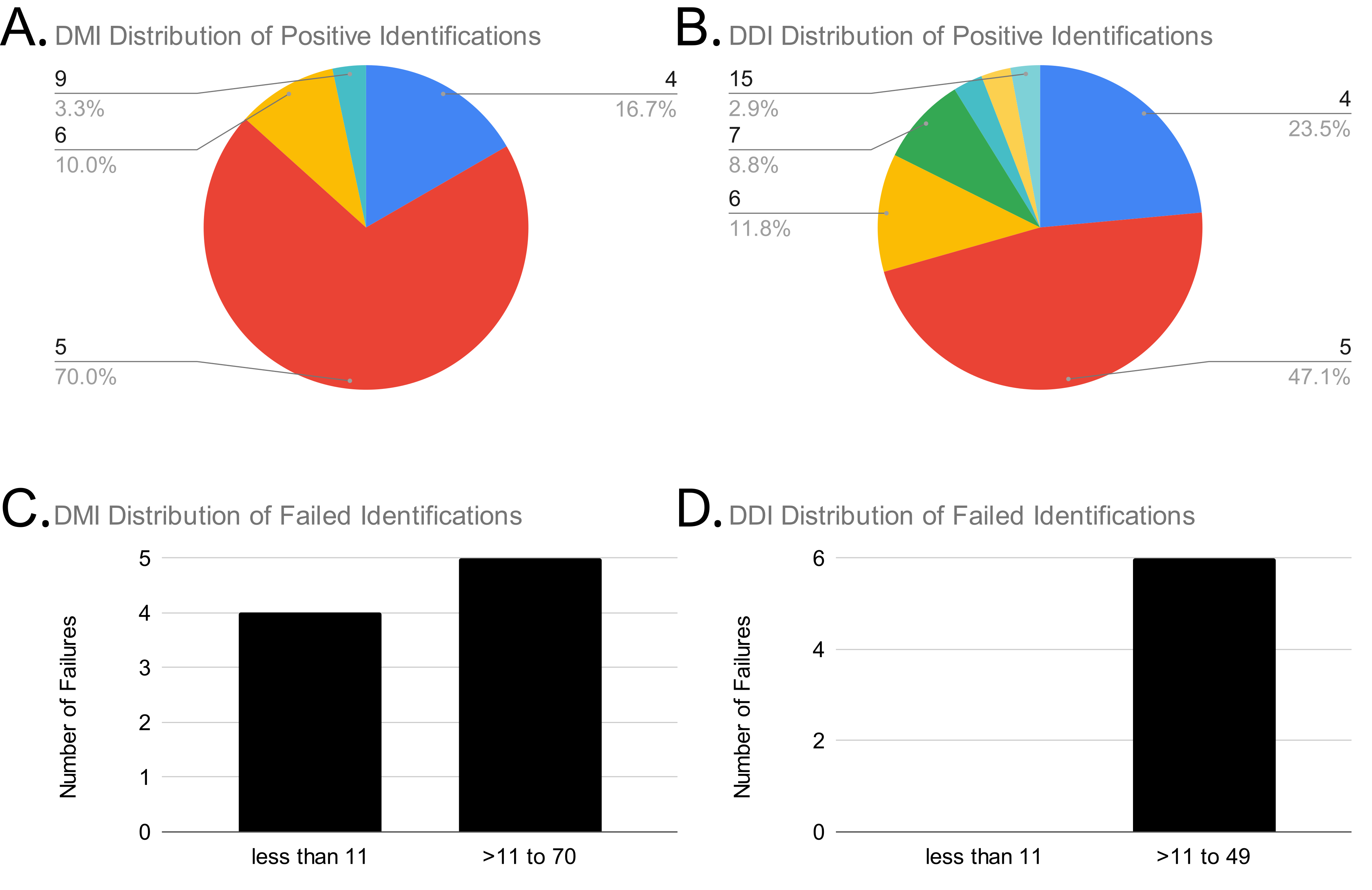

Supplement: S1 Fig — Contact filter distance was varied from 3 to 15 angstroms between alpha carbons in integer steps. Panels A and B) Distances at which correct interprotein interfaces were detected. Correct interfaces were determined from crystal structures. Each slice of the pie chart is identified with the contact filter distance at which the DMI or DDI was identified. Below the contact distance is the percent of the test cases that were detected at this filter setting. No interactions were detected below 4 angstroms and no correct interactions were observed above 15 angstroms. Panel A shows the distribution for correct DMI identifications and Panel B shows the distribution for correct DDI identifications. Panels C and D) For some models, the correct DMI or DDI was not identified. On the X axis are the ranges, in angstroms, at which an interaction was detected. Failures were distributed over ranges shown. DMI failures occurred at contact filter settings of 4, 5, 5, 7, 21, 33, 39, 64, and 70 angstroms. DDI failures occurred at 14, 16, 18, 22, 27, and 49 angstroms. (TIFF) [file pcbi.1013062.s001.tiff]
